# Study of Natural Health Product Adverse Reactions (SONAR): Active Surveillance of Adverse Events Following Concurrent Natural Health Product and Prescription Drug Use in Community Pharmacies

**DOI:** 10.1371/journal.pone.0045196

**Published:** 2012-09-28

**Authors:** Sunita Vohra, Kosta Cvijovic, Heather Boon, Brian C. Foster, Walter Jaeger, Don LeGatt, George Cembrowski, Mano Murty, Ross T. Tsuyuki, Joanne Barnes, Theresa L. Charrois, John T. Arnason, Candace Necyk, Mark Ware, Rhonda J. Rosychuk

**Affiliations:** 1 Complementary and Alternative Research Program (CARE) for Integrative Health and Healing, Department of Pediatrics, Faculty of Medicine and Dentistry, University of Alberta, Edmonton, Alberta, Canada; 2 School of Public Health, University of Alberta, Edmonton, Alberta, Canada; 3 Department of Pediatrics, Faculty of Medicine and Dentistry, University of Alberta, Edmonton, Alberta, Canada; 4 Department of Medicine, Faculty of Medicine and Dentistry, University of Alberta, Edmonton, Alberta, Canada; 5 Women and Children’s Health Research Institute, Edmonton, Alberta, Canada; 6 Leslie Dan Faculty of Pharmacy, University of Toronto, Toronto, Ontario, Canada; 7 Department of Clinical Pharmacy and Diagnostics, University of Vienna, Vienna, Austria; 8 Faculty of Medicine, University of Ottawa, Ottawa, Ontario, Canada; 9 Health Canada, Ottawa, Ontario, Canada; 10 Department of Laboratory Medicine and Pathology, Faculty of Medicine and Dentistry, University of Alberta, Edmonton, Alberta, Canada; 11 School of Pharmacy, Faculty of Medical and Health Sciences, University of Auckland, Auckland, New Zealand; 12 School of Pharmacy, Curtin Health Innovation Research Institute, Perth, Australia; 13 Department of Biology, Faculty of Science, University of Ottawa, Ottawa, Ontario, Canada; 14 Faculty of Pharmacy and Pharmaceutical Sciences, University of Alberta, Edmonton, Alberta, Canada; 15 Alan Edwards Pain Management Unit, McGill University Health Centre, Montreal, Quebec, Canada; University of British Columbia, Canada

## Abstract

**Background:**

Many consumers use natural health products (NHPs) concurrently with prescription medications. As NHP-related harms are under-reported through passive surveillance, the safety of concurrent NHP-drug use remains unknown. To conduct active surveillance in participating community pharmacies to identify adverse events related to concurrent NHP-prescription drug use.

**Methodology/Principal Findings:**

Participating pharmacists asked individuals collecting prescription medications about (i) concurrent NHP/drug use in the previous three months and (ii) experiences of adverse events. If an adverse event was identified and if the patient provided written consent, a research pharmacist conducted a guided telephone interview to gather additional information after obtaining additional verbal consent and documenting so within the interview form. Over a total of 112 pharmacy weeks, 2615 patients were screened, of which 1037 (39.7%; 95% CI: 37.8% to 41.5%) reported concurrent NHP and prescription medication use. A total of 77 patients reported a possible AE (2.94%; 95% CI: 2.4% to 3.7%), which represents 7.4% of those using NHPs and prescription medications concurrently (95%CI: 6.0% to 9.2%). Of 15 patients available for an interview, 4 (26.7%: 95% CI: 4.3% to 49.0%) reported an AE that was determined to be “probably” due to NHP use.

**Conclusions/Significance:**

Active surveillance markedly improves identification and reporting of adverse events associated with concurrent NHP-drug use. Although not without challenges, active surveillance is feasible and can generate adverse event data of sufficient quality to allow for meaningful adjudication to assess potential harms.

## Introduction

Recent national surveys in North America, Australia and Europe suggest that more than half of the population uses dietary supplements, also known as natural health products (NHPs) or complementary medicines, including herbs, vitamins, minerals and other supplements. [1]–[6] In developing countries, use is even higher; in Africa for example, 80% of the population uses African Traditional Medicine, 90% of which is plant-based. [5].

NHPs are generally considered to be safe by the public, despite the growing evidence that this is not always the case. [1], [7]–[11] Particular caution is warranted when NHPs are used in combination with prescription medications due to the potential for interactions. [1] Of note, NHPs are frequently used by patients with chronic or recurrent conditions; these patients are also the most likely to be prescribed conventional medications. [12]–[14] Since the likelihood of an adverse event (AE), including drug interactions, increases with the number of medicinal products used, it is hypothesized that patients who concurrently use prescription medications and NHPs are therefore at greater risk for an AE than if they were using either product alone and represent a population of particular interest with respect to exploring the safety of NHPs. [10].

In most countries, the primary system of identifying post-marketing AE related to therapeutic products is passive surveillance (i.e., voluntary spontaneous reporting). [15] Increasingly, these passive surveillance systems are also used for collecting information about NHP-related AEs, as is the case in the United States, Canada, the United Kingdom, Australia, and Germany. [15]–[18] A strength of passive surveillance is its potential to identify AEs from large populations that are using products under real world circumstances, which should theoretically allow for the identification of new and/or rare adverse events. [16], [17].

Passive surveillance depends on voluntary reporting of adverse events by health care practitioners (and, in some cases, patients) and is known to be limited by substantial under-reporting with estimates suggesting that perhaps less than 1% of AEs are ever reported. [16]–[20] Patients may be even less likely to report AEs associated with NHPs as compared to those associated with conventional over-the-counter medications (OTC); some may not associate NHPs with causing harms or may not consider NHP-associated AEs important enough to report. [21], [22] Among health care providers, pharmacists have higher reporting rates, but like other health professionals, they too under-report suspected NHP AEs in comparison to those for conventional pharmaceuticals. [19], [23] This may be due to lack of awareness of their patients NHP use: studies in the UK and Australia have found that a majority of pharmacists does not ask customers about NHP use, including when receiving reports of suspected AEs associated with prescription medicines. [24], [25] Similarly, physicians are poor at reporting drug-related harms, and they do not routinely inquire about their patients’ NHP use. [19], [23], [26] Combined, these patient and health care provider factors suggest that passive surveillance has important limitations with respect to identifying NHP-related harms.

Another approach to investigating drug safety is active surveillance which “seeks to ascertain completely the number of adverse events via a continuous pre-organized process”. [27] Active surveillance methods are well-established for the collection of adverse event data following prescription medicine use, [28], [29] but its application to pharmacovigilance of NHPs is limited to date. Compared with passive surveillance, active surveillance collects adverse event reports (i.e., harmful or unintended health outcome which is not necessarily related to the use of a drug) and, thus, achieves increased reporting rates, often with more comprehensive, better quality reports. [27] A study designed to improve AE reporting in a primary care setting specializing in complementary and alternative medicine (CAM) demonstrated a 148% increase in reporting. [30] Similarly, Al-Tajir and Kelly found the incidence of adverse drug events associated with prescription medications detected through active surveillance was significantly higher (p<0.001) than for those reported spontaneously for both inpatients (3.592 vs. 0.068/100 patient days) and outpatients (0.299 vs. 0.022/100 patient visits). [31].

Other forms of active surveillance of adverse events include population-based administrative databases, such as British Columbia’s PharmaNet database, linked with hospitalizations and health care use, and other computerized health-record databases, such as the UK General Practice Research Database. [32], [33] However, these databases do not record NHP use and, therefore are not useful at present for pharmacovigilance of this class of products. [34].

While not an active surveillance study, community pharmacists in a retrospective cross-sectional study in England were asked to describe reports of suspected adverse reactions associated with herbal medicines that they had identified or received (e.g. from customers/patients) over the previous year. [24], [34] In total, among 818 respondents, 44 such reports were described (one per 19 pharmacists). [24], [34] By contrast, in the national pilot scheme in England for community pharmacist involvement in passive surveillance that ran at the same time as the cross-sectional study, among 3200 participant pharmacists, only 4 such reports were submitted through passive surveillance (one per 800 pharmacists). [35].

Since many NHPs are purchased in pharmacies in North America, and since pharmacists are trained to recognize potential product-related adverse reactions, including drug interactions, pharmacies are an appropriate setting for an active reporting model for NHP-related AEs. [19], [21], [36]–[38] The *S*tudy *O*f *N*atural health product *A*dverse *R*eactions (SONAR) was a multi-centre study assessing a community pharmacy-based active surveillance system to identify NHP-drug interactions.

## Methods

Research Ethics Board approval for this study was obtained from both the University of Alberta and the University of Toronto.

Because there are quality issues with (at least, some) NHPs that have an impact on safety, NHP-related harms can only be fully interpreted with laboratory analysis of the implicated product(s) in question. Therefore, we designed a two phase study, comprising active surveillance and a causality assessment process that included laboratory analysis to assess for contamination, adulteration and NHP-drug interactions.

### Phase I: Active Surveillance

The objective of this study was to use active surveillance in ten community pharmacies in the Greater Toronto Area in Ontario, Canada to identify AEs related to concurrent NHP-prescription medication use. Participating pharmacies were selected using convenience sampling to represent a range of pharmacy types including: independent and chain pharmacies, pharmacies with and without a special focus on NHPs, and outpatient hospital pharmacies.

Participating pharmacy staff members were instructed to ask all individuals collecting prescriptions for themselves or their child about NHP use and concurrent NHP-prescription drug use in the previous three months and the presence of any experiences of AEs by using a standardized data form developed specifically for this purpose. (Table 1) NHPs were defined using the Health Canada definition as any medicinal product with active ingredients found in nature and suitable for over-the-counter use including vitamins, minerals, herbal remedies, homeopathic medicines, traditional medicines, probiotics, amino acids and fatty acids. [39] If no NHP use was identified in Question 1, no further questions were asked. Three months was selected as the sampling frame as this represents a typical time period between filling prescriptions for chronic conditions to avoid repeated sampling from the same individuals. The pharmacy staff made no causality assessment of a reported AE; all information was passed on for further assessment. If an AE was identified, the patient was asked to provide written consent to share his/her contact information with a research pharmacist (KC) to schedule a follow-up telephone interview. Verbal consent was obtained at the onset of the phone interview. The interview collected details required for causality assessment and information about the patient’s health state, all products taken (prescription, over-the-counter (OTC) medications, and NHPs) including brand and dose, hospitalizations and recent medication changes. Patients were asked to describe what they felt may have caused their AE; subsequently, independent experts adjudicated the cases regardless of patient opinion. The patient interview form was developed specifically for this purpose based on the reporting requirements of Health Canada, the US Food and Drug Administration, and the European Medicines Agency. [40]–[42] A copy of the patient interview form is available from the corresponding author upon request.

**Table 1:** Pharmacy standardized patient screening form.

Screening Questions:
**1.** Are you currently using *NHPs* such as herbs, vitamins or other supplements, or have you used such products in the previous 3 months? (If NO, no additional questions. If YES, proceed to #2).
**2.** Have you taken a prescription medication while also taking a *NHP* in the previous 3 months? (If NO, no additional questions. If YES, proceed to #3).
**3.** Have you experienced any unexpected or undesirable effects during the last 3 months? (If NO, no additional questions. If YES, proceed to #4).
**4.** Would you agree to be contacted by a pharmacist from our coordinating centre to conduct a telephone interview? (If NO, no additional questions. If YES, provide study information sheet and document patient’s name, phone number, best time to call and patient’s signature)

In order to prepare participating pharmacy staff members, a training session was scheduled for each pharmacy to present the study protocol and address questions or concerns. To further assist pharmacists who might be faced with patients’ questions or concerns regarding NHP-drug combinations and their effects, a tool for quick identification of NHP-drug interactions was developed and distributed to the participating pharmacists. [43] Also, every pharmacy was given a textbook on NHPs as a reference. [44] Finally, participating pharmacies were visited regularly by study team members to address any issues and assist with implementation of the data collection process.

### Phase II: Causality Assessment and Laboratory Analysis

AE reports were developed based on telephone interviews and assessed by our adjudicating committee, a three member panel consisting of one NHP expert, one clinical expert, and the committee chair, a clinician with expertise in pharmacology and NHPs. The adjudicating committee assessed AE reports based on the Naranjo algorithm, the Horn algorithm and World Health Organization (WHO) causality assessment criteria. [45]–[47] The adjudicating committee was asked to determine: (i) if there was a causal relationship between the AE and any product the patient was taking; (ii) if there was an NHP-drug interaction; and (iii) if laboratory analysis was required. (Table 2) Three laboratories were involved in evaluation of potential NHP-related harms: (i) NHP constituent assessment; (ii) adulterant/contaminant evaluation; and (iii) NHP-drug interaction evaluation (i.e. ability of NHP to inhibit cytochrome P450-mediated metabolism). For their analyses, the laboratories tested the actual products the patients were taking when the AE occurred, as well as additional lots of the products implicated to run comparative analyses.

**Table 2:** Algorithm to assess the need for suspect products to undergo laboratory analysis.

1. Laboratory evaluation for potential pharmacokineticor pharmacodynamic interaction of the NHPwith prescription medications	2. Assessment for potential adulterantsor contaminants within the product	3. Issues related to product quality, whetherheterogeneity or pharmacologicalactions of NHP components
Interactions between an NHP and drug assessed as“definite”, “probable” or “possible”	Causality of adverse events from NHP productalone classified as being “possible” or higher	Causality of adverse events from NHP productalone classified as being “possible” or higher
Unexpected increase or decrease in drug levels ortherapeutic effect of a previously stable drug; or difficulty inachieving stable therapeutic effect or drug level in a newlyinitiated drug	The NHP source was India, China or Mexico(indicating a higher likelihood of contamination)and causality for the report was classified asbeing “probable” or “possible”	
NHP-drug combination has been identified asyellow/orange/red (indicating a potential risk) in theNHP-drug interaction tool [41]	NHP which is known to be often adulteratedwith prescription drugs (e.g. NHPs for weightloss, muscle enhancement, sexual enhancementmarketed as having anti-inflammatory properties)and is causality for the report is classified as being“probable” or “possible”	
The product is well known to cause pharmacokineticand/or pharmacodynamic interactions with drugs/NHPs.(Products with high index of suspicion: atazanavir,betanaphthoflavone, carbamazepine, clarithromycin, dexamethasone, digoxin, efavirenz, fluoxetine, fluvoxamine,gemfibrozil, indinavir, insulin, isoniazid, itraconazole,ketoconazole, lithium carbonate, methylcholanthrene,modafinil, nafcillin, nefazodone, nelfinavir, nevirapine,norethindrone, omeprazole, oxcarbazepine, paroxetine,pentobarbital, phenobarbital, phenytoin, pioglitazone,prednisone, quinidine, rifabutin, rifampin, ritonavir, saquinavir,secobarbital, telithromycin, theophylline, troglitazone, warfarin)		

### Statistical Analysis

Analysis focused on Phase I data where proportions by pharmacy and questionnaire version were calculated. A logistic regression model with only an intercept term was used to provide the weighted average proportion for each outcome and the associated 95% confidence intervals (CIs). [48], [49] SAS version 9.1 was used for all analyses. [50].

## Results

### Phase I: Active Surveillance

Participating pharmacies included five chain and four independent pharmacies, of which three self-proclaimed they specialized in NHPs, as well as one hospital outpatient pharmacy. The mean number of prescriptions filled daily in the participating sites was 367 (range 70–1800). All pharmacy staff members agreed to participate in the study (n = 29), including 17 pharmacists (10 of whom were pharmacy managers or owners), 1 pharmacy intern, 2 pharmacy students, 6 pharmacy technicians, 2 nutritionists, and 1 store manager (who was not a pharmacist).

**Figure 1:**
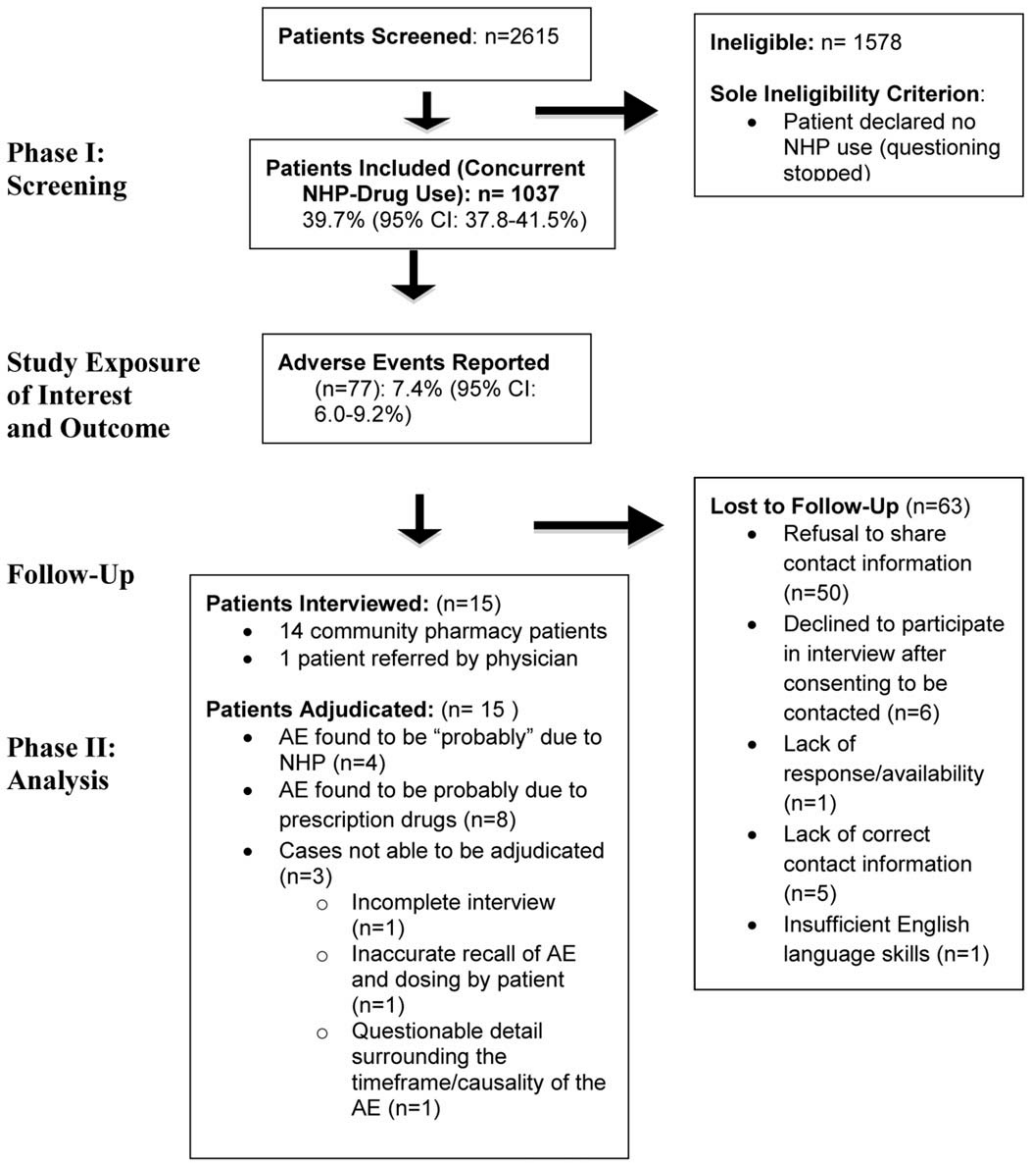
Flow diagram of Phase I and II Results.

The first pharmacy was enrolled in March 2008, and data collection was completed after a total of 112 pharmacy weeks. Overall, 2615 patients were screened. Of these, 1037 reported using NHPs and prescription medications concurrently (weighted proportion = 39.7%; 95% CI: 37.8% to 41.5%). Among those using NHPs and prescription medicines concurrently, 77 patients reported experiencing AEs (weighted proportion = 7.4%; 95% CI: 6.0% to 9.2%). (Tables 3 and 4) Approximately one-third (36%; n = 27) of patients who reported an AE originally agreed to be contacted for the follow-up telephone interview. However, of these, only 15 patients subsequently were interviewed, of whom 14 were recruited through the participating pharmacies and one was referred to the study by her physician. Thirteen patients were not interviewed for several reasons including: refusal to participate in telephone interview (n = 6); lack of correct contact information (n = 5); inability to adequately communicate in English via telephone (n = 1); or lack of response despite multiple contact attempts (n = 1). Detailed information was therefore collected in 23% of the suspected AE cases. Figure 1 provides a flow diagram of numbers of participants progressing through the study.

**Table 3:** Proportion of concurrent use of NHP and prescription medication by pharmacy.

Pharmacy	Participants (*n_i_*)	Concurrent Use (*c_i_*)	Concurrent Use (*p_i_*)
ON01	439	174	39.6%
ON02	189	67	35.5%
ON03	502	232	46.2%
ON04	249	132	53.0%
ON05	340	72	21.2%
ON06	137	44	32.1%
ON07	11	4	36.4%
ON08a	25	7	28.0%
ON08b	168	74	44.1%
ON09	211	60	28.4%
ON10	344	171	49.7%
**Total**	**2615**	**1037**	**39.7%**

**Table 4:** Proportion with adverse events for those concurrently taking NHP and prescription medication (MED) by pharmacy.

Pharmacy	Concurrent Use (*n_i_*)	Adverse Events (*c_i_*)	Adverse Events (*p_i_*)
ON01	174	10	5.8%
ON02	67	1	1.5%
ON03	232	16	6.9%
ON04	132	6	4.6%
ON05	72	0	0.0%
ON06	44	1	2.3%
ON07	4	0	0.0%
ON08a	7	1	14.3%
ON08b	74	10	13.5%
ON09	60	11	18.3%
ON10	171	21	12.3%
**Total**	**1037**	**77**	**7.4%**

### Phase II: Causality Assessment and Laboratory Analysis

Following causality assessment of detailed telephone interviews, 4 of the 15 were adjudicated as “probably” related to patients’ NHP(s) use based on adjudication with the Naranjo and WHO assessment scales. [45], [47] In 3 out of 4 cases, the adjudicating committee referred the products for laboratory analysis to evaluate for probable NHP-drug interactions based on assessment using the Horn scale. [46] Due to space constraints, only brief summaries of the four case reports are discussed below; detailed reports are published elsewhere for 2 of the 4 reports. One of these was categorized as a serious AE because the patient required hospitalization.

Overall, 11 of the 15 reports were considered unrelated to NHP use. Eight of these cases were attributed to known adverse drug reactions; examples including: muscle pain with atorvastatin; nausea with extended-release tramadol XR (with positive dechallenge); several generalized allergic reactions, e.g., urticarial rash. In these cases, the NHP(s) had been used for a significant period of time before the occurrence of the AE, while the suspect drugs were changed or added in close proximity to the onset of symptoms. Thus, there was a strong temporal association with the suspect drugs, rather than the NHP(s). The remaining 3 cases were inaccessible/unclassifiable due to an incomplete interview (n = 1), inaccurate recall of the AE including dosing (n = 1) and questionable details around the timeframe of the AE (n = 1). Summary details of case reports with causality assessed as “probably” related to NHP use follow. Table S1 summarizes data obtained from all telephone interviews and their adjudication.

#### Case 1

A 19 year old male patient with depression, neuropathic pain and delayed sleep phase disorder presented after addition of melatonin to his current regimen of citalopram, nortriptyline, and oxycodone with what he described as severe sedation. Samples of the patient’s melatonin, together with samples of another lot of the same product and of other melatonin products, were all tested to show cytochrome P450 (CYP) 2C19 inhibitory activity in vitro. Because citalopram is metabolized by CYP2C19, our study team suspects a combined pharmacodynamic/pharmacokinetic mechanism of interaction which has not previously been documented. (See File S1).

#### Case 2

A 38 year old female patient with hormone disorder experienced fatigue, nervousness, heart palpitation, rash, and muscle twitching following the addition of two blended herbal products to her existing regimen comprising a multivitamin containing green tea extract and a compounded progesterone cream. The two blended multi-constituent products were screened for their ability to inhibit CYP-mediated metabolism. Both inhibited CYP2C19 and CYP2D6 activity *in vitro* and may, therefore, have interacted with the constituents of the multivitamin or progesterone products. [51].

#### Case 3

A 71 year old female with hypertension and hypothyroidism reported symptoms of severe flushing after the addition of niacin 1000mg once daily to her regimen of enalapril 10 mg and a compounded medication for her thyroid. The flushing slowly improved once her physician ordered her to discontinue the niacin and completely cleared after a period of a few days. Lab analysis was not recommended for this case, as flushing was recognized as a common side effect of niacin.

#### Case 4

A 53 year old female patient with asthma and who also had symptoms related to menopause and depression/anxiety was hospitalized for jaundice and fatigue and diagnosed with hepatic necrosis. The patient was taking 13 different health products, including several NHPs for weight loss and several pharmaceuticals. Several products of both groups have been found in an in-depth literature review to have hepatotoxic properties (conjugated linoleic acid, GHR®, NutriMin C®, Softcap Fish Oil®, varenicline, venlafaxine). This case illustrates the potential dangers of uncontrolled intake of multiple NHPs in combination with prescription drugs without medical supervision. [52].

## Discussion

In this study, we present one of the first prospective community-based active surveillance studies to assess NHP related AEs and NHP-drug interactions. Although not without its challenges, active surveillance in pharmacies is feasible, and markedly increases both the number and quality of AE reports in comparison to passive surveillance. [53]–[56] When comparing our results to data derived from passive surveillance, we found that the Health Canada AE database (Canada Vigilance) has a total of 1544 (median = 233.5, range = 144 in 2004 to 442 as of December 31, 2009) AE reports associated with NHP use over a five year period from passive surveillance of over 30 million Canadians, which results in a median incidence rate of approximately 0.0008% (range = 0.0005% in 2004 to 0.0015% in 2009). [57] Given that one third of Canadians report taking NHP and prescription medications concurrently, the median incidence rate of AE reports is approximately 0.0023% (range = 0.0014% in 2004 to 0.0044% in 2009). [4] These values are far lower than AE rates reported by individuals taking NHPs and drugs concurrently in the SONAR study (7.4%; 95% CI: 6.0% to 9.2%), suggesting that active surveillance improved AE reporting rates substantially. It is important to note that the population screened in our study may represent a different population than those screened through passive surveillance. In particular, patients being screened in a pharmacy may be more likely to take prescription medications; since we hypothesized this may increase the risk of potential AEs, this was the population of greatest interest to our study team.

Of note, without adjudication, it is not clear how many of the AE reports from either active or passive surveillance are causally linked to NHP use. In its surveillance system, Health Canada applies a signal detection process to the incoming AE data associated with all health products, including NHPs, and causality assessment is conducted accordingly. However, often the quality of AE reports is poor and important details allowing for proper causality assessment are missing. [58].

Studies from several countries report that NHP use is extremely common. While several have assessed concurrent use with prescription medications, [59]–[61] few have reported on the frequency of NHP-drug interactions associated with concurrent use. In the 2002 Canadian National Health Population Survey, 21% of respondents had used both prescription medications and NHPs in the previous year and 28.4% of those were using combinations with potentially harmful interactions. [62] Data collected from the 2002 United States Health and Diet Survey revealed that 4% of NHP users reported at least one adverse event over the previous year. [59] In addition, those NHP users reporting an AE were more likely to be taking prescription medications and NHPs concurrently than those not reporting an AE (74% vs. 58% respectively). [59] The population surveyed in this study may differ in important ways from our urban community pharmacy population which may partially explain the differing AE rates.

While pharmacy-based active surveillance improved the quantity and quality of AE reports, numerous challenges were encountered. These included challenges in finding pharmacies willing to participate and pharmacy staff members not screening as many patients as expected. The most effective strategies to counter these challenges were involvement of pharmacy staff with previous research experience, involving recent graduates and students in data collection, as well as close contact and frequent visits to the sites to ensure implementation of the study protocol. This pilot program involved pharmacies/pharmacists who volunteered to participate in NHP safety research and, therefore, may not be representative of all pharmacies/pharmacists in Canada. On the other hand, this was an entirely new concept – for pharmacists to take responsibility for adverse event reporting (and with our results, perhaps more pharmacists will be interested in NHP adverse events).

Potential sampling bias also extended to the consumers screened. Only those sufficiently healthy enough to collect their or their child’s prescriptions from the pharmacy were screened. Limiting screening to outpatient community pharmacies minimizes the ability to identify current AEs requiring hospitalization or past AEs resulting in hospitalization or even death. In addition, since the pharmacy sites screened fewer patients than was originally expected, it is possible the choice of who to screen was biased in some way. Pharmacy participants stated that patient screening depended on workload. However this was difficult to quantify: limited data were provided to us on the number of prescriptions filled by each pharmacy during the study period, but this number is likely to be much lower than the number of consumers served. Recall bias with respect to patient reports of AEs was another limitation. When possible, hospital records and laboratory data were collected to ensure the highest level of accuracy. Reassuringly, our study found rates of NHP use and NHP-drug concurrent use similar to those reported by others, suggesting that our sampling frame was acceptable. [2], [63]–[65] In this study, the AEs were identified by patients in an out-patient setting rather than by clinicians or other health professionals, which is more typical of some active surveillance designs. While this limited the data available to investigate the AE, such as obtaining drug/NHP plasma concentrations, our approach helped overcome several major obstacles in detecting NHP-related harms, namely lack of inquiry about NHP use, lack of inquiry about experiences of harms, and lack of reporting, even if harms are identified.

The community setting offered a key strength to our design: SONAR screened a comparatively large sample of patients who were using NHPs under real-world conditions. The study resulted in the development of a practical tool (the NHP-drug interaction tool), [43] that has been published and has since been in use by a number of clinicians who have confirmed its usefulness. To our knowledge, SONAR is the first to systematically capture NHP-drug interactions by identifying clinically relevant harms first, coupled with basic science investigation to examine the plausible mechanism. More often in the literature, NHP-drug interactions have been posited on theoretical grounds but their clinical relevance remains unknown, as these harms have not been identified in clinical practice. [66]–[68] Although follow-up interviews were difficult to schedule, the quality of data obtained allowed meaningful adjudication: all cases deemed to be “probably” NHP related, and referred for lab analysis for evaluation of NHP-drug interaction, had confirmatory lab findings. Strength of our design was the opportunity to investigate the mechanism of action of NHP AEs through laboratory research, promoting the detection of novel clinically relevant NHP-drug interactions. It is important to note that with a greater degree of AE reporting comes a greater possibility of loss to follow-up, and this is an important limitation in conducting active surveillance. Further studies should investigate the sensitivity and specificity of the adjudication process, as well as methods to improve follow-up in active surveillance of NHP-related harms.

Serious harms tend to be rare, and their detection is extremely challenging. Harms assessment is a multistage process: product use must be disclosed and discussed; product-related harms must be consistently included in the differential diagnosis; suspected harms must be reported in sufficient quantity and quality for signal detection to occur. Active surveillance offers means to promote discussion and allows for meaningful adjudication, and, therefore offers an important contribution to patient safety and pharmacovigilance. It is difficult to determine causality of an AE if potential drug interactions, product authenticity adulterants/contaminants and NHP components are not investigated simultaneously. [69] The paucity of literature regarding the risks involved with NHP use is not enough reason to consider these products “safe”. [70] Only systematic data collection and analysis will provide the necessary evidence to consider an NHP safe, particularly in the context of concurrent use with prescription medications.

In practice, although clinicians and patients can tolerate certain degrees of uncertainty surrounding effectiveness (i.e. a therapeutic trial or “try-and-see” approach), they are understandably less tolerant of uncertainty surrounding safety. We found 7% of those concurrently using NHPs with medications report AE, with 4/15 (26.7%; 95% CI: 4.3% to 49.0%) probably due to NHP use; whether or not this is interpreted as low or high depends on one’s perspective.

Within Canada, provincial electronic health databases exist to facilitate active surveillance by capturing adverse event information relating to prescription drugs as they occur. [32] Although these databases may have the ability to capture NHP and over-the-counter drug data, this is not widely known and thus they are therefore still limited in their ability to actively capture AEs within this scope of product use. [34].

Our study provides a novel method for investigating harms relating to NHPs to assess causality and provide clinical evidence for future patients. Active surveillance improved detection and reporting, and the rigorous investigation of detected harms generates clinical evidence to allow for practice-based change, promoting patient safety. Additional work is needed to establish if active surveillance is cost effective. According to a Health Council of Canada publication, the annual operating costs for the Marketed Health Products Directorate was $23.6 M for the year ending March 31, 2010 and is anticipated to be $30.5 M for the year ending March 31, 2012; MHPD has confirmed that approximately 10% of their budget is spent on NHP surveillance activities. [71] (See File S2) Our research team was able to start the first NHP AE active surveillance program for approximately 1% of the MHPD NHP budget. Further research is required to determine the potential economic impact of implementing active surveillance on a wider scale.

In conclusion, active surveillance for NHP-related AEs in community pharmacies is feasible. Further investigation of its potential contributions to assessment of NHP safety is warranted. Future research includes the addition of NHP AE screening within already developed provincial electronic health databases, and the use of both active surveillance and causality assessment in high risk populations, such as those seen in hospital subspecialty clinics. There is also an opportunity to test the utility of active surveillance in other countries that regulate NHPs, as it may prove informative for both NHP and drug-related adverse events based on real-world use. The limitations of passive surveillance have been well documented, but the potential advantages of active surveillance have yet to be fully explored.

## Supporting Information

Table S1 **Case summaries for all detailed patient interviews.**
(DOCX)

File S1 **Cvijovic et al. 2011 Unpublished manuscript supporting Case 1.**
(DOC)

File S2 **MHPD Memo (February 23, 2012) supporting Health Canada passive surveillance cost data.**
(PDF)
