# Supplementary material for: Study of Natural Health Product Adverse Reactions (SONAR): Active Surveillance of Adverse Events Following Concurrent Natural Health Product and Prescription Drug Use in Community Pharmacies
Source: PLoS One. 2012 Sep 28;7(9):e45196. doi: 10.1371/journal.pone.0045196 (PMC3461007; doi:10.1371/journal.pone.0045196)
Supplement: Table S1 — Case summaries for all detailed patient interviews. (DOCX) [file pone.0045196.s001.docx]

**Table S1. Case summaries for all detailed patient interviews.**

| **Cases assessed as “probably” due to NHP(s)** | | | | | | | |
| --- | --- | --- | --- | --- | --- | --- | --- |
| **Case** | **Patient Age** | **Patient Sex** | **Prescription**  **drugs** | **NHP products** | **Adverse Event** | | **Adjudication Result** |
| 1 | 19 | M | citalopram, nortriptyline, oxycodone | melatonin | Severe sedation | | Probable |
| 2 | 38 | F | progesterone cream | FemMed, Thyrosense, multivitamin | Fatigue, nervousness, heart palpitation, rash, and muscle twitching | | Probable |
| 3 | 71 | F | enalapril, compounded thyroid medication | niacin | Flushing | | Probable |
| 4 | 53 | F | budesonide- formoterol, estradiol, progesterone, lorazepam, varenicline, venlafaxine | acidophilus with bifidus, CLA, GHR (growth hormone), MSM, NutriMin C, fish oils, vitamin D | Hepatic Necrosis | | Probable |
| **Cases not assessed as “probably” due to NHP(s)** | | | | | | | |
| 5 | 64 | M | phenytoin, leuprolide, acetylsalicylic acid, hydrocortisone | vitamin C, vitamin D | Headache, pain, weight changes, depressive symptoms. | Unlikely | |
| 6 | N/A | N/A | levothyroxine | N/A | Double vision, dizziness, headache | Unassessable: Interview incomplete due to loss of contact | |
| 7 | 71 | F | alendronate, progesterone cream | calcium, vitamin D | Reflux, stomach upset | Unlikely | |
| 8 | 52 | F | nitrofurantoin, venlafaxine | multivitamin, vitamin B complex, 100% Alaskan Salmon & Fish oil blend, EFA, homeopathic medicine (name N/A) | Anaphylactic reaction | Unclassifiable: inaccurate recall of timing around event | |
| 9 | 65 | F | atorvastatin, metformin | multiple (>25) | Urticaria | Unclassifiable: inaccurate recall of doses and timing around event | |
| 10 | 50 | F | progesterone 6% cream, hydrocholor-thiazide, | vitamin C | Sweating | Unlikely | |
| 11 | 75 | F | atorvastatin, amiloride, atenolol, acetylsalicylic acid | calcium, vitamin D, multivitamin | Muscle pain in left leg | Unlikely | |
| 12 | 55 | F | tramadol XL, hydroxychlor-iquine | multivitamin, vitamin D | Headache and nausea | Unlikely | |
| 13 | 61 | F | moxifloxacin | calcium, vitamin D | Rash | Unlikely | |
| 14 | 66 | M | dipyridamole-acetylsalicylic acid, ramipril-hydrocholorothiazide, bupropion, rosuvastatin | vitamin E, vitamin C, glucosamine/ chondroitin | Headache, nausea, flu-like symptoms | Unlikely | |
| 15 | 58 | F | clopidogrel, simvastatin, amlodipine, atenolol, vinpocetine | co-enzyme Q10, vitamin D, calcium | Headache | Unlikely | |
