# Supplementary material for: Study of Natural Health Product Adverse Reactions (SONAR): Active Surveillance of Adverse Events Following Concurrent Natural Health Product and Prescription Drug Use in Community Pharmacies
Source: PLoS One. 2012 Sep 28;7(9):e45196. doi: 10.1371/journal.pone.0045196 (PMC3461007; doi:10.1371/journal.pone.0045196)

**“Natural” may not mean safe: Challenges assessing potential harms associated with polypharmacy including multiple natural health products**

Kosta Cvijovic^1,2^, Heather Boon^1^, Walter Jaeger^2^, Sunita Vohra^3, 4^

^1^Leslie Dan Faculty of Pharmacy, University of Toronto, Toronto, Ontario; ^2^Department of Clinical Pharmacy and Diagnostics, University of Vienna, Vienna, Austria; ^3^CARE Program, Department of Pediatrics, Faculty of Medicine and School of Public Health, University of Alberta, Edmonton, Alberta; ^4^School of Public Health, University of Alberta, Edmonton, Alberta

**Corresponding Author:**

Sunita Vohra MD FRCPC MSc
Professor
Dept of Pediatrics
Faculty of Medicine and School of Public Health
University of Alberta
 
8B19 - 11111 Jasper Ave
Edmonton General Hospital
Edmonton, Alberta
Canada
T5K 0L4
 
Phone: (780) 342-8592
Fax: (780) 342-8464
Email: [CARE@med.ualberta.ca](https://webmail.utoronto.ca/imp/message.php?index=10)
Website: www.pedcam.ca <<http://www.pedcam.ca/>>
              www.care.ualberta.ca <<http://www.care.ualberta.ca/>>

| **Key points** |
| --- |
| - Natural health products such as herbs, vitamin and amino acids may interact with prescription drugs with potentially serious consequences - Natural health products are complex chemical entities that make assessment of harms challenging - A growing number of natural health products have documented hepatotoxic properties - Clinicians should include routine inquiry about complementary and alternative medicine use as part of their history-taking |

**Introduction**

Almost 75% of Canadians now use natural health products (NHPs) such as herbs, vitamins and other supplements (1) and 57% report using NHPs concurrently with prescription medications.(2) Polypharmacy has previously been identified as increasing the risk of patients experiencing adverse events, (3) but most focus has been on adverse events caused by concurrent use of multiple prescription drugs. This case highlights the potential dangers of combining multiple NHPs and drugs and the challenges associated with investigating harms when they occur.

**Case report**

A 53 year old 158 cm, 57 kg female asthmatic patient with symptoms related to menopause and depression/anxiety experienced fatigue and jaundice starting approximately Christmas 2008 which worsened until March 2009. She reported regular intake of 3-4 beers and/or a few glasses of wine daily prior to the onset of the adverse event. She was hospitalized in March 2009 for jaundice and discharged four days later with a tentative diagnosis of cirrhotic liver disease based on an abdominal ultrasound. She was re-hospitalized (at a different hospital) twelve days later, because of increasing severity of jaundice and fatigue. She reported discontinuing her alcohol intake after her first discharge. On examination she was mildly confused (consistent with hepatic encephalopathy), had poorly formed spider nevi and had moderate ascites. She was found to be hepatitis B immune, and negative or normal for the following: hepatitis C virus, ceruloplasmin, alpha-1 antitrypsin, anti-mitochondrial antibodies (AMA), antinuclear antibody, and antismooth muscle antibody. Results of laboratory investigations from both hospitalizations can be found in Table 1. Sodium, potassium, chloride, total CO_2_, haemoglobin, platelets, white blood cells, calcium, creatinine, random glucose, lipase, magnesium, and urea were in the normal range. Key abnormalities were alanine transaminase (ALT) 755 and alkaline phosphatise (ALP) 273 during the first admission as well as bilirubin 441 during the second admission. Abdominal ultrasound was suggestive of cirrhosis, showed no evidence of hepatic or portal venous thrombosis, and no biliary dilatation. A Doppler ultrasound showed no evidence of hepatic or portal venous thrombosis. A transjugular liver biopsy showed submassive necrosis without features of alcoholic hepatitis. A biopsy slide along with its interpretation can be found in Figure 1. Autoimmune hepatitis was considered but upon reviewing her medication profile, an adverse reaction to one or more drugs was considered the most likely explanation.

The patient had been taking seven NHPs, four prescription medications and one non-prescription medication in the three months preceding the adverse event. A detailed list of the products is provided in Tables 2 (pharmaceuticals) and 3 (NHPs). The patient described starting all the NHPs shortly before the onset of the adverse event (December 2008) except for the human growth hormone product (GHR®) which was started in October 2008 for weight loss and was discontinued in December 2008. All NHPs and pharmaceuticals were discontinued on admission in March except budenoside / formoterol fumarate dihydrate and venlafaxine. A graphic timeline outlining the start and stop dates of the products, as well as the onset of the event and the hospitalization, can be found in Figure 2. The patient obtained many of the NHPs online and did not inform her health care professionals about taking them prior to the event.

During the second hospital visit, the patient was managed with furosemide 40 mg orally initially, reduced to 20 mg once daily, spironolactone orally 25 mg increased to 50 mg once daily, and lactulose 20 g daily, as well as two sennoside tablets at bedtime. Prednisone 40 mg daily was started given the severity of her hepatic illness and the liver biopsy findings. The patient was discharged from her second hospital stay after ten days. Two weeks later, the patient was reviewed by the treating physician and found not to have any asterixis and to be less jaundiced but still having obvious icterus. The patient was clinically improved. During a follow-up in September 2009, it was observed that the patient’s condition has improved further (see Table 1).

**Discussion**

This case was obtained through the Pharmacy *S*tudy *O*f *N*atural Health Product *A*dverse *R*eactions (SONAR) which is a multi-center study assessing the feasibility of a community pharmacy-based active surveillance system to identify adverse reactions related to NHPs. (4) In addition to assessment by the SONAR adjudication committee, a review of the possible hepatotoxic properties of the ingredients of all the products ingested by this patient (including their excipients if known) was conducted using MedLine, EMBASE, Natural Medicines Comprehensive and e-CPS databases from inception to August 2009 (limited to articles available in English). Keywords were the names of the ingredient(s) or excipients of the products combined with “liver” and ”hepato*”.

The results of this search are summarized in Table 4. Several compounds found in the NHPs the patient was taking have reports of hepatotoxicity in the literature; however, many are based on animal or *in vitro* data, and the few clinical reports generally lack precise dosage information.(5-14) It is possible that many of the NHPs ingested by the patient may have contributed to the event Both venlafaxine and varenicline have been reported to cause hepatotoxicity in patients, especially in those with underlying liver conditions (15-17) which suggests they may have played a role in the event. The patient’s alcohol intake may also be an important factor in this case.

Based on the clinical case and the results of the literature review, lab analysis of the NHPs was considered necessary in order to determine whether adulterants or contaminants were involved. Samples were obtained from the patient for all NHPs she had in her possession: conjugated linoleic acid (CLA), methylsulfonylmethane (MSM), NutriMin C®, and the fish oil product. Of note, the patient did not have any of the human growth hormone (GHR®) product and it was no longer available in Canada at the time of lab testing. Additional samples from the product brands described above but different lot numbers from those provided by the patient were also purchased for evaluation. The samples were analyzed by gas chromatography/mass spectrometry, as well as ultraviolet (UV) manual screening for steroids; these are methods developed in-house at the University of Alberta Hospital. The samples were not found to contain any adulterants or contaminants.

Peak abnormalities for the liver function tests demonstrate the severity, and clearly show the hepatocellular nature, of the injury in this patient. Given the wide range of both pharmaceuticals and NHPs ingested, combined with the alcohol intake, it is impossible to identify a specific cause. This case highlights the challenges associated with investigating serious harms that may be associated with concurrent use of prescription medicines, NHPs, other non-prescription medicines and alcohol. The clinical timing of the symptoms suggests possible involvement of the NHPs, but this cannot be confirmed despite in-depth investigation of the case. Another alternative possibility would be autoimmune hepatitis; however, the patient did not have the usual serum markers to support the latter diagnosis.

The human growth hormone product (GHR®) is of particular interest to this case because Health Canada has issued a warning about it, citing risks associated with unsubstantiated health claims, hyperthyroidism, and possible interactions and allergic reactions. (18) GHR is not currently authorized for sale in Canada. As usual procedure, Health Canada advises that all suspected adverse events associated with any health product be reported to Health Canada’s Canada Vigilance Program. (19)

The public generally considers NHPs to be safe, despite the fact that pharmacodynamic and pharmacokinetic interactions with conventional pharmaceutical medicines have been reported. (20) This perception of safety appears to make patients less likely to report suspected adverse events related to NHPs than to conventional medicines. (21, 22) Coordinated national surveillance initiatives like the SONAR study might help alleviate this problem by increasing both the quantity and quality of reports (23). This case illustrates that, when multiple products with potential to cause adverse events in the same physiological area are combined, it is very hard to determine the interactive potential and main source of the event, underlining the importance to obtain more and better information on the safety and interactive potential of both drugs and NHPs.

In summary, as the majority of Canadians use NHPs and about one third report using more than 3 NHPs concurrently (1), clinicians should include inquiry regarding NHP use as a routine part of their medical history taking and be sure to report any suspected adverse events to the Canada Vigilance program. NHP-drug interactions should be included in the differential diagnosis when expected treatment effects do not occur, or there are unexplained harms. Many NHPs may indeed be safe and new Canadian regulations which require safety information on the label of licensed products will hopefully help consumers make informed treatment choices; however, when multiple NHPs are combined with multiple prescription and over-the-counter (OTC) products, significant harms can occur and caution is warranted.

**Acknowledgment:** The SONAR team thanks the University of Alberta Department of Laboratory Medicine and Pathology, Division of Anatomic Pathology, for providing us with the biopsy image. In addition, we would like to thank the additional members of the SONAR study team: Brian C. Foster(Faculty of Medicine, University of Ottawa, Ottawa, Ontario; Health Canada, Ottawa, Ontario), Lauren Girard (CARE Program, Department of Pediatrics, Faculty of Medicine and School of Public Health, University of Alberta, Edmonton, Alberta), Don LeGatt (Department of Laboratory Medicine and Pathology, Faculty of Medicine and Dentistry, University of Alberta, Edmonton, Alberta), Mano Murty (Health Canada, Ottawa, Ontario), Duc Vu (Health Canada, Ottawa, Ontario), Ross T. Tsuyuki (Department of Medicine, University of Alberta, Edmonton, Alberta; School of Public Health, University of Alberta, Edmonton, Alberta), Joanne Barnes (School of Pharmacy, Faculty of Medical and Health Sciences, University of Auckland, Auckland, New Zealand)

**Resource Box:**

Adverse Event Reporting (including for natural health products):

Med Effect Canada:

<http://www.hc-sc.gc.ca/dhp-mps/medeff/report-declaration/index-eng.php>

Natural health product-drug interaction tool:

http://www.cpjournal.ca/archive/1913-701X/142/5/pdf/i1913-701X-142-5-e1.pdf

General Information about Natural Health Products

*Freely Accessible*

Health Canada’s information about natural health products: <http://www.hc-sc.gc.ca/dhp-mps/prodnatur/index-eng.php>

Natural Health Products Directorate of Health Canada:

<http://www.hc-sc.gc.ca/ahc-asc/branch-dirgen/hpfb-dgpsa/nhpd-dpsn/index-eng.php>

CAMline: Evidence-based Reviews of Natural Health Products

[www.CAMline.ca](http://www.CAMline.ca/)

National Centre for Complementary and Alternative Medicine:

<http://nccam.nih.gov/>

*Subscription Required:*

Natural Standard, The Authority on Integrative Medicine:

<http://www.naturalstandard.com/>

Natural Medicines Comprehensive Database:

[http://www.naturaldatabase.com](http://www.naturaldatabase.com/)

*Books*

Barnes J, Anderson LA, Phillipson JD. Herbal medicines. 3rd ed. London (UK): Pharmaceutical Press; 2007.

Boon H and Michael Smith. 55 Most Common Medicinal Herbs 2^nd^ Ed. Toronto: Robert Rose; 2009

**References**

1. Ipsos Reid. Baseline natural health product survey among consumers. (Accessed September 21^st^, 2009, at <http://www.hc-sc.gc.ca/dhp-mps/alt_formats/hpfb-dgpsa/pdf/pubs/eng_cons_survey-eng.pdf>)
2. Singh SR and Levine MAH. Potential interactions between pharmaceuticals and natural health products in Canada. Journal of Clinical Pharmacology 2007;47:249-258
3. Haider SI, Johnell K, Thorslund M, Fastbom J. Trends in polypharmacy and potential drug-drug interactions across educational groups in elderly patients in Sweden for the period 1992 - 2002. *International Journal of Clinical Pharmacology and Therapeutics* 2007;45(12):643–653
4. Cvijovic K, Boon H, Brulotte J, et al. Pharmacy study of natural health product adverse reactions (SONAR): Piloting an active surveillance model in community pharmacies. Pharmaceutical Biology 2009;47 (Supplement 1):21 (published abstract)
5. Rauen U, Klempt S, De Groot H. Histidine-induced injury to cultured liver cells, effects of histidine derivatives and of iron chelators. *Cellular and Molecular Life Sciences* 2007;64(2):192-205
6. Scarna A, Gijsman HJ, McTavish SF et al. Effects of a branched-chain amino acid drink in mania. *British Journal of Psychiatry* 2003;182(3):210-3
7. Garlick, PJ. The nature of human hazards associated with excessive intake of amino acids. *Journal of Nutrition* 2004;134(6):1633S-9S
8. Hamid S, Rojter S, Vierling J. Protracted cholestatic hepatitis after the use of prostata. *Ann Intern Med* 1997;127(2):169-170
9. Ramos R, Mascarenhas J, Duarte P, Vicente C, Casteleiro C. Conjugated linoleic acid-induced toxic hepatitis: First case report. *Digestive Diseases & Sciences* 2009;54(5):1141-3
10. Leekumjorn S, Wu Y, Sum AK, Chan C. Experimental and computational studies investigating trehalose protection of HepG2 cells from palmitate-induced toxicity. *Biophysical Journal* 2008;94(7):2869-83
11. Fallon MB, Boyer JL. Hepatic toxicity of vitamin A and synthetic retinoids. *Journal of Gastroenterology & Hepatology* 1990;5(3):334-42
12. Leo MA, Lieber CS. Alcohol, vitamin A, and beta-carotene: Adverse interactions, including hepatotoxicity and carcinogenicity. *American Journal of Clinical Nutrition* 1999;69(6):1071-85
13. Kitano M, Watanabe D, Oda S et al. Subchronic oral toxicity of ubiquinol in rats and dogs. *International Journal of Toxicology* 2008;27(2):189-215
14. Ritskes-Hoitinga J, Verschuren PM, Meijer GW et al. The association of increasing dietary concentrations of fish oil with hepatotoxic effects and a higher degree of aorta atherosclerosis in the ad lib.-fed rabbit. *Food & Chemical Toxicology* 1998;36(8):663-72
15. Sencan I, Sahin I, Ozcetin A. Low-dose venlafaxine-associated liver toxicity in chronic hepatitis [letter]. Ann Pharmacother. 2004;38(2):352–3
16. Horsmans Y, De Clereq M, Sempeux C. Venlafaxine-associated hepatitis [letter]. Ann Intern Med. 1999;130(11):944
17. Franck AJ, Sliter LR. Acute Hepatic Injury Associated with Varenicline in a Patient with Underlying Liver Disease. *Ann Pharmacother.* 2009;43(9):1539-1543
18. Health Canada warns consumers not to use human growth hormone drug called GHR-15 (Accessed October 28^th^, 2009, at <http://www.hc-sc.gc.ca/ahc-asc/media/advisories-avis/_2005/2005_55-eng.php>)
19. Health Canada’s Canada Vigilance Database (Accessed October 28^th^, 2009, at <http://www.hc-sc.gc.ca/dhp-mps/medeff/databasdon/index-eng.php>)
20. Baily DG, Dresser GK. Natural products and adverse drug interactions. *Canadian Medical Association Journal* 2004;170(10):1531-2
21. Barnes J, Mills SY, Abbot NC, Willoughby M, Ernst E. Different standards for reporting ADRs to herbal remedies and conventional OTC medicines: face-to-face interviews with 515 users of herbal remedies. *British Journal of Clinical Pharmacology* 1998;45(5):496-500
22. Walji R. Reporting adverse drug reactions associated with herbal products: consumer, health food store personnel and pharmacist perspectives. PhD dissertation, University of Toronto, 2008.
23. Community-based active surveillance of adverse events associated with use of natural health products (published abstract on the homepage of the Canadian Patient Safety Institute) <http://www.patientsafetyinstitute.ca/English/research/cpsi-cihr/priorityOperatingGrants/Pages/Vohra.aspx> (accessed March 14, 2010)

**Table 1: Laboratory investigations of the patient**

| March 2009 | | |
| --- | --- | --- |
| ALT | 755* | Normal range: 8-56 |
| ALP | 273* | Normal range: 42-98 |
| Bilirubin | 281 | Normal range: 3.4-22 |

| April 2009 | | |
| --- | --- | --- |
| Bilirubin | 441* | Normal range: 3.4-22 |
| AST | 524 | Normal range: 6-34 |
| ALT | 317 | Normal range: 8-56 |
| ALP | 247 | Normal range: 42-98 |
| Albumin | 30 | Normal range: 3.5-5.5 |
| INR | 1.7 | Normal range: 0.9-1.2 |
| PTT | 36 | Normal range: 18-28 |

*peak abnormalities

| September 2009 | | |
| --- | --- | --- |
| ALP | 156 | Normal range: 42-98 |
| ALT | 39 | Normal range: 8-56 |
| Bilirubin | 52 | Normal range: 3.4-22 |
| Albumin | 40 | Normal range: 3.5-5.5 |
| INR | 1.1 | Normal range: 0.9-1.2 |

Table 2: Pharmaceutical medications the patient reported to have taken

| 1. NAME | 1. STRENGTH/ DOSE | 1. INTAKE FREQUENCY | 1. THERAPY START | 1. THERAPY END | 1. INDICATION |
| --- | --- | --- | --- | --- | --- |
| 1. Budesonide/ formoterol fumarate dihydrate | 1. 200 μg/ 2. 6 μg | 1. BID | 1. approximately 2004 | 1. ongoing | 1. Asthma |
| 1. Estradiol | 1. 50 μg/24 hours | 1. 2-weekly patch | 1. approximately 2007 | 1. March 2009 | 1. Menopause |
| 1. Lorazepam | 1. 1 mg | 1. once daily | 1. approximately 2006 | 1. March 2009 | 1. Anxiety, smoking cessation |
| 1. Progesterone | 1. 100 mg | 1. unknown | 1. approximately 2007 | 1. March 2009 | 1. Menopause |
| 1. Varenicline | 1. 0.5 mg | 1. once daily | 1. December 2008 | 1. March 2009 | 1. Smoking cessation |
| 1. Venlafaxine | 1. 150 mg | 1. unknown | 1. approximately 2004 | 1. ongoing | 1. Depression/ anxiety |

**Table 3: Natural health products the patient reported to have taken**

| 1. BRAND NAME | 1. STRENGTH/ DOSE | 1. INTAKE FREQUENCY |
| --- | --- | --- |
| 1. Acidophilus with Bifidus | Lactobacillus rhamnosus 50% / 3.0 billion cfu, Lactobacillus casei 30% / 1.8 billion cfu, Lactobacillus acidophilus 10% / 0.6 billion cfu, *Bifidobacterium longum* 10% / 0.6 billion cfu | 1. TID |
| 1. Conjugated Linoleic Acid (CLA) | Tonalin 1000 mg per 1 capsule, CLA (74-82%), Oleic Acid 10-20%, Palmitic Acid 6%, Stearic Acid 3% | TID |
| 1. GHR® 2. (human growth hormone) | Anterior Pituitary porcine source 20 mg, Hypothalamus 5 mg, Amino acid blend 300 mg, Lysine, Histidine, Arginine, Asparatic acid, Threonine, Serine, Glutamic acid, Leucine, Phenylalanine, Panax ginseng 20 mg, Proline, Glycine, Alanine, Valine, Methionine, Isoleucine, Tyrosine, Phylosterol complex 10 mg, Beta sitosterol, Campesterol, Stigmasterol, Soy phosphatide serene 40%, Phosphatidyl choline, Phosphatidyl ethanolamine, Phosphatidylinositol | 1. unknown |
| 1. MSM | 1. Methosulfonylmethane 1000 mg | 1. 1-3 capsules 2. daily |
| 1. NutriMin C® | Vitamin A (Retinyl palmitate) 188 mcg, Vitamin C (Calcium ascorbate) 15 mg, Vitamin E (d-alpha-tocopheryl acetate) 7.5 mg, Flax seed oil (70% alpha linolenic acid) 520 mg), Alpha lipoic acid 3.75 mg, Ubidecarenone (Ubiquinone-10) 3.75 mg | 1. 2 capsules BID |
| 1. Softcap Fish Oil® | Fish body oil (From Sardine, Salmon, Anchovy, Sprat, and Herring) 1000 mg, EPA (Eicosapentaenoic acid) 180 mg, DHA (Docosahexaenoic acid) 120 mg | 1. BID |
| 1. Vitamin D | Vitamin D 1000 IU (Cholecalciferol) | 1. once daily |

**Table 4: Potential Hepatoxocity of Natural Health Products and Prescription Drugs**

| **Product Name** | **Ingredient** | **Hepatotoxic Potential** |
| --- | --- | --- |
| Acidophilus with Bifidus | *Bifidobacterium longum, Lactobacillus acidophilus, Lactobacillus casei, Lactobacillus rhamnosus* | No evidence of hepatotoxicity found in the literature |
| Conjugated Linoleic Acid | Conjugated Linoleic Acid | Clinical case, no dose provided (9) |
|  | Palmitic Acid | In vitro toxicity(10) |
|  | Oleic Acid, Stearic Acid | No literature found |
| GHR® | Histidine | In vitro toxicity(5-7); no adverse effects in humans at doses less than 4.5g/day (7) |
|  | Methionine | Animal and clinical evidence of liver toxicity(7); no adverse effects as does less than 5g/day; 30g iv resulted in liver dysfunction(7) |
|  | Panax Ginseng | Clinical case of 1 patient with protracted cholestatic hepatitis after use of combination product (Protstata®) containing ginseng; ginseng dose not reported(8) |
|  | Anterior Pituitary, Arginine, Aspartic Acid, Beta Sitosterol, Campesterol, Glutamic Acid, Glycine, Hypothalamus, Isoleucine, Leucine, Lysine, Phenylalanine, Phosphatidylcholine, Phospathidylethanolamine, Phosphatidylinositol, Phytosterol, Proline, Serine, Soy phosphatide serene, Stigmasterol, Threonine, Tyrosine, Valine | No evidence of hepatotoxicity found in the literature (7) |
| MSM | Methylsulfonylmethane | No evidence of hepatotoxicity found in the literature |
| NutriMinC ® | Vitamin A (retinyl palmitate) | Clinical evidence of hepatotoxicity with doses of 2.5-3 times daily requirements for 10+ years or 70-80 times daily requirements for 1 year(11);; lowest dose reported associated with liver cirrhosis is 7500 RE(25000 IU) for 6 years (12) |
|  | Vitamin C | Most literature suggests that vitamin C has a protective effect, but radical ascorbate may cause problems |
|  | Ubidecarenone | Animal evidence (13) |
|  | Alpha lipoic acid, Flax seed oil, Vitamin E | No evidence of hepatotoxicity found in the literature |
| Softcap Fish Oil® | Fish body oil | Animal evidence (14) |
|  | DHA, EPA | No evidence of hepatotoxicity found in the literature |
| Vitamin D | Vitamin D | No evidence of hepatotoxicity found in the literature |
| Varenicline | Varenicline | Case report of hepatic injury with dose of 0.5mg daily escalating to to 1 mg twice day; underlying alcoholic liver disease(17); 3 case reports of hepatotoxicity when used concomitantly with other medications in the Canada Vigilance database |
| Venlafaxine | Venlafaxine | Case report of liver toxicity in patient with history of liver disease at dose of 37.5 mg/day (15) Additional cases at higher doses (15,16) |

^1^The product suggests there is an essential amino acid blend, but cysteine and tryptophan are not listed on the ingredient list

Excipients of all products were reviewed, no reports of hepatotoxicity were found for these

**Figure 1: Liver core biopsy shows marked hepatocyte loss; Hematoxylin and Eosin**


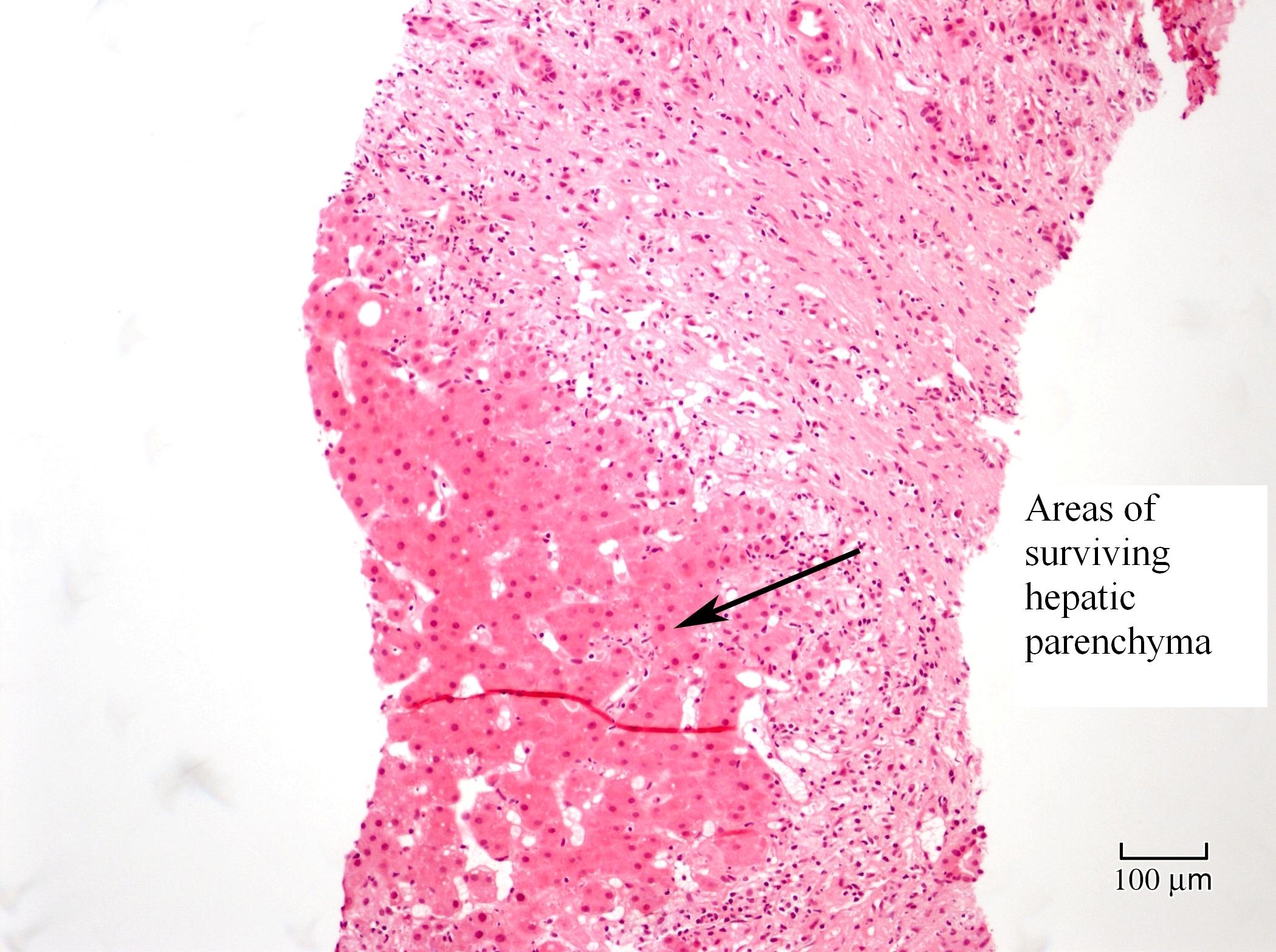


**Figure 2: Timeline of drug/natural health product exposures, adverse reaction onset and hospitalization**


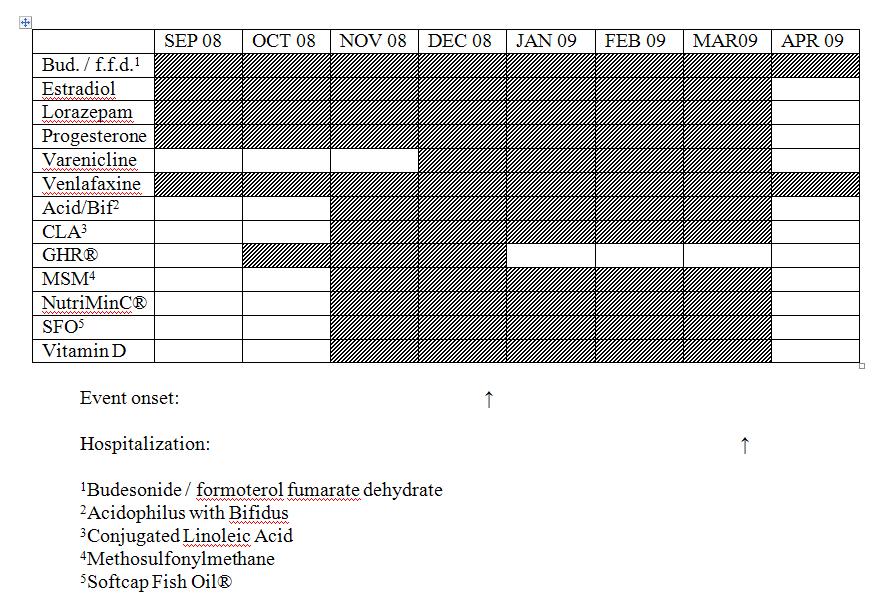

Supplement: File S1 — Cvijovic et al. 2011 Unpublished manuscript supporting Case 1. (DOC) [file pone.0045196.s002.doc]
