# Supplementary material for: Study of Natural Health Product Adverse Reactions (SONAR): Active Surveillance of Adverse Events Following Concurrent Natural Health Product and Prescription Drug Use in Community Pharmacies
Source: PLoS One. 2012 Sep 28;7(9):e45196. doi: 10.1371/journal.pone.0045196 (PMC3461007; doi:10.1371/journal.pone.0045196)
Supplement: File S2 — MHPD Memo (February 23, 2012) supporting Health Canada passive surveillance cost data. (PDF) [file pone.0045196.s003.pdf]

Marketed Health Products Directorate
A.L. 0702C
OTTAWA, Ontario
K1A 0K9

February 23, 2012

12-102291 - 803

Sunita Vohra
Director, CARE Program for Integrative Health and Healing
University of Alberta
svohra@ualberta.ca

Dear Sunita Vohra:

Re: Cost Information

This letter is in response to your email of January 26, 2012 in regards to cost information and adverse reaction statitics.

You inquired about an estimate of *what percentage of Marketed Health Products Directorate (MHPD) budget looks at Natural Health Products adverse events (AE) vs. all the other activities of MHPD*. Approximately 10% of the MHPD budget is spent in health product surveillance activities related to Natural Health Products.

You inquired for an *estimate on how many drug AE were reported, and how many "other" AE were reported during the same interval*. Here is a chart to provide you with the annual comparison as requested:

Number of Post-market Domestic Adverse Reaction (AR) Reports* by Year

Year	NHPs	Pharmaceuticals	Others**	Total
2008	382	14513	6144	21039
2009	516	18301	7244	26016
2010	677	22104	10140	32921
2011***	608	25304	12430	38342

*The Canada Vigilance Program receives reports for both initial and follow-up information concerning suspected adverse reactions.
**"Others" includes reports of e.g., biotechnology products, biologics, radiopharmaceuticals, cells, tissues and organs.

.../2

This report is produced with the information available at that point in time. New or additional information may be received by the Canada Vigilance Program at a later date which will be reflected in the statistics at that time.

Adverse reactions to health products are considered to be suspicions, as a definite causal association often cannot be determined. Spontaneous reports of ARs cannot be used to estimate the incidence of ARs because ARs remain underreported and patient exposure is unknown.

We hope this information is helpful to you.

Marketed Health Products Directorate
Email: mhpd_dpsc.public@hc-sc.gc.ca
